# Microbial Contamination and Food Safety Aspects of Cassava Roasted Flour (“Rale”) in Mozambique

**DOI:** 10.3390/microorganisms13010168

**Published:** 2025-01-15

**Authors:** Andreia Massamby, Su-lin L. Leong, Bettina Müller, Lucas Tivana, Volkmar Passoth, Custódia Macuamule, Mats Sandgren

**Affiliations:** 1 Department of Molecular Sciences, Uppsala BioCentrum, Swedish University of Agricultural Sciences, P.O. Box 7051, 750 07 Uppsala, Sweden; andreia.massamby@slu.se (A.M.); su-lin.leong@slu.se (S.-l.L.L.); bettina.muller@slu.se (B.M.); volkmar.passoth@slu.se (V.P.); 2 Faculty of Agronomy and Forestry Engineering, Eduardo Mondlane University, Maputo P.O. Box 257, Mozambique; lucastivana@yahoo.co.uk; 3 Centre of Excellence in Agri-Food Systems and Nutrition, Eduardo Mondlane University, Maputo P.O. Box 257, Mozambique; 4 Faculty of Veterinary Sciences, Eduardo Mondlane University, Maputo P.O. Box 257, Mozambique; custodia.macuamule@uem.mz

**Keywords:** cassava roasted flour, “rale”, food quality, food safety, microbial contamination, microbial diversity, Mozambique

## Abstract

Cassava is an important staple food that contributes to the food security of small-scale Mozambican farmers. In southern Mozambique, cassava roots are usually processed into cassava roasted flour, locally known as “rale”. The handling and processing practices connected to “rale” production may introduce microbial contamination. We assessed the microbial contamination of “rale” processed in local farmers’ associations and consumed either locally or sold in rural markets. Microbial sampling was carried out both during the warmer rainy and cooler dry seasons, and microorganisms of relevance for food safety and fermentation were enumerated. The results revealed variation in terms of microbial diversity in all stages of cassava root processing. In samples collected in the warmer rainy season, molds, lactic acid bacteria, general aerobic bacteria and *Bacillus* spp. were isolated, whereas in samples collected in the cooler dry season, other groups of microorganisms such as yeasts and *Staphylococcus aureus* were present. *Wickerhamomyces anomalus*, *Rhodotorula mucilaginosa*, *Pichia exigua*, *Meyerozyma caribbica* and *Torulaspora delbrueckii* were the most frequent yeast species found within the cassava processing stages. Aflatoxin-producing molds were observed infrequently in this study, and only at low counts, thus, the risk for aflatoxin contamination appears to be low. The results obtained from the Illumina 16S rRNA gene sequencing can be considered a complementary technique to the plating methods relied on in this study. From a food quality and safety point of view, this staple food does not appear to pose a high risk for foodborne disease.

## 1. Introduction

Cassava (*Manihot esculenta Crantz*) is an important staple food in most tropical regions worldwide and represents a source of nourishment especially in Africa, Asia, and South America [1,2]. Africa is considered the continent with the largest cassava production, where this crop is cultivated in around 40 countries. Nigeria is the largest producer, harvesting more than 59 Mt of fresh cassava roots annually [3,4,5,6]. In Mozambique, cassava is ranked as the most important staple food, followed by maize [7]. At least 97% of small-scale Mozambican farmers select cassava as a main production crop, due to its ability to grow in different ranges of climate and altitudes, and its tolerance to a wide variety of soils, diseases and drought when compared to other agricultural crops [2,8,9].

This subsistence crop is important for food security at several levels, both for government and rural families—it has potential to increase farmers’ incomes, and reduce rural and urban poverty levels [10,11,12]. It is considered an attractive and low-risk crop for African farmers, being produced with family labor using simple hand instruments [13]. Cassava also holds a great promise for feeding Africa’s growing population, as a readily available and cheap staple food for low–income rural households [12].

Producing nutritional and safe food products from cassava is a challenge for many reasons. Fresh cassava roots have a very short shelf-life of 1–3 days after harvest, limiting food security [8,14]. After harvesting, fresh cassava roots deteriorate rapidly due to a complex biochemical and physiological process, known as postharvest physiological deterioration, PPD. The rapid PDD reduces both the shelf life and quality attributes of cassava roots [15]. Furthermore, despite its nutritional value, cassava contains antinutrients such as phytates, tannins (phenolics), oxalates, nitrates/nitrites, and saponins. These compounds can be toxic and hinder the absorption of certain nutrients [13,16,17]. Some bitter cassava varieties contain high levels of toxic cyanogenic compounds (cyanogenic glycosides) in edible parts. These cassava varieties can represent a source of intoxication for consumers if not prepared properly [13]. The use of adequate processing techniques can reduce both the antinutrients and the cyanogenic glycoside levels in cassava varieties, resulting in better nutrient quality, higher levels of vitamins, especially the B group, essential amino acids, and improvement in protein digestibility [13,18].

In Africa, the processing techniques for dried fermented cassava products such as cassava flour, often lead to low quality products since they are not usually protected during the drying process from environmental contamination including the action of animals and pests. As a result, fouling products, exposure to microorganisms, mycotoxin formation and contamination by pathogens can be observed [1]. Records of aflatoxin contamination by *Aspergillus flavus*, *Aspergillus nomius*, and *Aspergillus parasiticus* have previously been reported in cassava processed products such as cassava roasted fermented flour from Malawi and Zambia [19]. On the other hand, another study in Tanzania concluded that samples of cassava flour collected immediately after the drying process did not show any aflatoxin contamination [20]. Likewise, contamination of stored cassava flour by *A. flavus*, *Aspergillus niger*, *Rhizopus stolonifer*, *Mucor racemosus* and *Fusarium oxysporum*, has also been reported in Nigeria [21].

In southern Mozambique, cassava is commonly processed to produce cassava roasted flour, also known as “rale”, a traditional food consumed by families. Most of the ”rale” production in the country is handmade, using simple tools and non-motorized equipment, by rural producers for their own consumption or sale. The industrial production of “rale” in Mozambique is limited. However, there are some cassava processing associations that benefit from the use of specialized and more advanced equipment. Their products are destined for sale in small, open-air or rural markets in the regions, including in larger cities. The cassava roasted flour processing chain starts with the reception of the cassava roots, followed by washing, peeling, chopping, pressing, sieving, and finally, roasting of the final product. In the southern region of Mozambique, there are 18 identified cassava processing farmers associations which produce “rale”, of which 5 are in the Gaza province and 13 in the Inhambane province. The “rale” produced in these associations is intended for consumption by the association’s members and for sale in the local markets.

The processing of cassava roots to “rale” appears to be simple; however, it is necessary that hygienic aspects are controlled at all stages to avoid contamination by microorganisms that can compromise the quality of the final product and its safety for consumers [22]. Few studies have been carried out to deeply evaluate the microbiological safety and quality of this traditional fermented cassava product. The present study aimed at surveying relevant microbes as indicators of hygienic quality existing (a) within the whole chain of processing cassava in the main processing unit (production unit called “*Unit J*” in this study); (b) in “rale” produced in different cassava processing units; and (c) in “rale” sold in rural markets. Our results show how the microbial contamination can affect the food quality and safety of the cassava derivatives produced, consumed and sold in southern Mozambique based on microbiological and molecular approaches.

## 2. Materials and Methods

### 2.1. Description of Study Area

The cassava sampling took place in the Gaza (24°54′02.7″ S; 33°57′37.5″ E) and the Inhambane (24°39′06.0″ S; 34°36′27.0″ E) provinces, in the southern part of Mozambique and at two different occasions: November 2020 and August 2021. From a climatic point of view, what differentiates the two climate seasons in Mozambique is the amount of rain that falls during these seasons. Mozambique has two seasons: a rainy season which normally lasts from November to April, and a dry season between May and October.

The temperature remains relatively stable throughout the year with differences of just a few degrees between the seasons and between day and night. Maximum temperatures vary between 24 °C and 30 °C along the south coast, with the hottest months between December and February. The same happens with minimum temperatures ranging between 14 °C and 22 °C, with June and July having the coldest temperatures. The annual precipitation varies from 800 mm to 900 mm per year [23].

### 2.2. Sampling Procedure for Assessment of Indicator Microbes for Hygiene Quality

#### 2.2.1. Steps of Casava Processing Within *Unit J* and Sampling

The microbial contamination at different stages of processing cassava (from roots to cassava roasted flour) was assessed in the main cassava processing association unit, hereafter called *Unit J*. This unit is considered a model for cassava processing, including the application of food and hygiene practices during the “rale” processing.

**Figure 1:**
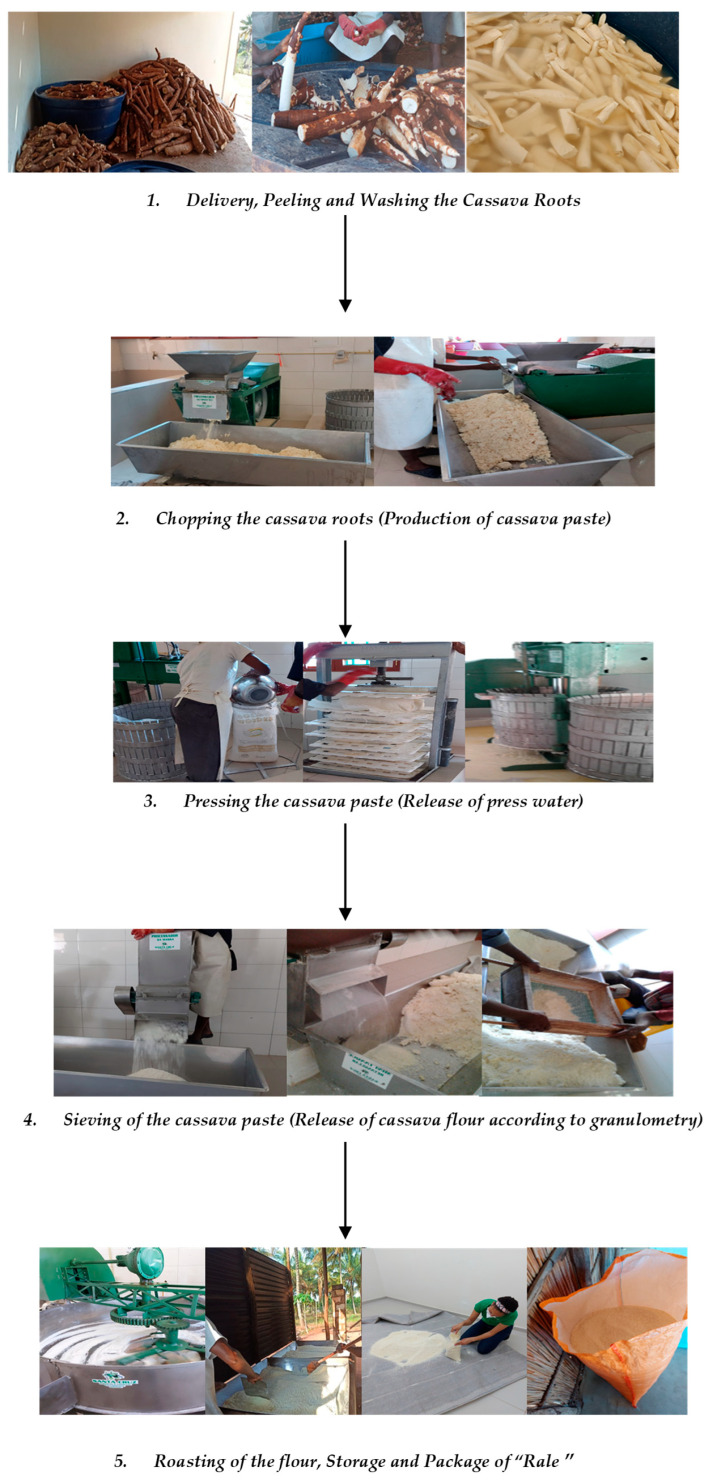
Stages of processing cassava within *Unit J*.

The cassava processing started with manual cassava harvesting at cassava farmers’ fields of *Unit J*. After harvest, the roots were transported to the association unit by animal-drawn carts or trucks. Upon arrival, cassava tubers were discharged directly onto a plastic sheet or net on the ground, where the association members proceeded with the peeling process. This was carried out outdoors and manually, without any washing or sanitizing step included. The peeled cassava roots were washed once by hand in big plastic basins containing tap water mixed with sodium hypochlorite to remove the impurities and soil residues. The washed and sanitized cassava roots were placed in clean plastic basins and thereafter, 4 stages of processing were followed (Figure 1).

The first stage of cassava processing is called “*Chopping*”. This was performed using a gasoline-powered chopper. The chopped cassava mass was placed into clean raffia bags. After chopping the roots, the “*Pressing*” stage was carried out using a manually driven mechanical press for no more than 24 h. The raffia bags with chopped cassava mass were placed into the pressing machine. From this point, the press water was released, and the pressing stage was finished. The cassava pressed mass was taken out from the raffia bags and placed in big metal containers for sampling, and the press water was collected and stored in clean plastic bottles.

After pressing, the cassava mass was introduced into a gasoline-powered grater for disaggregation and reduction of agglomerates. This stage is called *“Grating”*. The “*Sieving*” process consisted of the screening of the cassava mass in a polyethylene net with wooden borders to separate the small homogenous particles from non-grated pieces and fibrous material.

Finally, the “*Roasting*” of the resulting processed cassava mass was either carried out outdoors (November 2020) or indoors (August 2021). This was due to the season and the installation of a new roasting machine at *Unit J*. The drying and cooling of “rale” was carried out on top of a tarpaulin on the floor of a closed storage room for approximately 7–15 days to reduce as much as possible the humidity left in the product. After cooling, the “rale” was placed in raffia bags, and when necessary, it was packed in plastic bags of 1 kg each for further distribution.

Approximately 500 g of casava material was randomly collected in triplicates for microbial assessment at each stage of the processing procedure, respectively: *Chopping*, *Pressing* (including the *Press water*), *Sieving* and *Roasting*, both during rainy and dry seasons.

#### 2.2.2. Sampling Procedure for Assessment of Indicator Microbes for Hygiene Quality in “Rale” Produced in Different Cassava Processing Units

To assess the microbial contamination in “rale” processed and stored in cassava processing units, six cassava processing units located in Gaza and Inhambane provinces, including the reference *Unit J*, were selected for sampling collection, and these cassava processing units are hereafter called *Units V*, *W*, *P*, *Z*, and *C*. From each season, approximately 500 g of stored cassava roasted flour was collected in triplicate at each processing unit. The cassava processing units were selected considering the following criteria: having a considerable cassava processing activity and having been trained to keep good hygiene practices during cassava processing according to Mozambican’s Agricultural Authorities.

#### 2.2.3. Sampling Procedure for Assessment of Indicator Microbes for Hygiene Quality in “Rale” Sold in Different Rural Markets

The study of the microbial contamination of “rale” sold in rural markets in Mozambique included a total of five different markets located along the National Road Number 1 (EN1), hereafter called Markets *AM*, *GB*, *MK*, *ES*, and *MC*. The EN1 connects the Southern part of the country to the Centre and Northern regions of Mozambique. The markets were selected considering easy access and history/or tradition of selling “rale”. In each market, three vendors were randomly selected, and approximately 500 g of the “rale” was collected in triplicates, comprising a total of 15 unique “rale” vendors.

The samples collected at different stages of processing cassava at *Unit J* (Section 2.2.1) and the “rale” collected both in cassava processing units (Section 2.2.2) and rural markets (Section 2.2.3) were stored in sterile plastic bags and kept frozen (−20 °C) until transportation to the Department of Molecular Sciences, Swedish University of Agricultural Sciences (Uppsala) for microbial and molecular analyses.

### 2.3. Culture-Based Analyses of Indicator Microbes for Hygiene Quality

All chemicals and culture media were obtained from Merck KGaA, Darmstadt, Germany; Sigma-Aldrich Inc., St. Louis, MO, USA and Oxoid Ltd., Basingstoke, Hampshire, UK. About 25 g of cassava sample from each triplicate was aseptically transferred to Erlenmeyer flasks containing 225 mL with sterile peptone water (0.1% peptone, *w*/*v*) to dilute the samples. The casava samples were then homogenized for 120 s at normal speed using a Stomacher 400 Laboratory blender (Seward Medical, London, UK). These samples were serially diluted and poured or spread plated on relevant selection media for enumeration of viable counts as specified by the manufacturer to isolate the desired microbes. The following groups of microorganisms were screened for:

(a) Yeasts and Molds

Yeasts and Molds were isolated by surface plating in triplicates on Dichloran Rose-Bengal Agar plates supplemented with 0.1 g/L Chloramphenicol to inhibit bacterial growth [24,25]. The yeast plates were placed in an incubator at 25°C for 72 h. After incubation, approx. 50 yeasts colonies representative of various colony morphologies present were transferred to Yeast Peptone Dextrose Agar (20 g/L peptone, 10 g/L Yeast extract, 20 g/L glucose, 20 g/L agar) supplemented with 0.1 g/L Chloramphenicol, and incubated at 25 °C for 2–3 days. Mold plates were incubated at 25 °C for 7 days. After incubation, approx. 30 molds representing all observed colony morphologies were transferred and cultivated on Malt Extract Agar at 25°C for 3–7 days for identification.

(b) Lactic Acid Bacteria and Bacterial Indicators of Hygienic Quality

*Lactic Acid Bacteria* (LAB) were quantified on De Man Rogosa Sharpe Agar supplemented with 0.1 g/L Delvocide (active compound, natamycin; Gist-Brocades B.V., Delft, The Netherlands) to inhibit fungal growth. Plates were incubated anaerobically using a GasPackTM EZ system (Becton Dickinson; Sparks, MD, USA) at 30 °C for 48 h [26,27].

*Total bacterial Counts* (TBC) were enumerated by the pour plate method using Tryptone Glucose Yeast Extract Agar supplemented with 0.1 g/L Delvocide to suppress fungal growth. Plates were incubated at 30 °C for 3 days [25,28].

*Enterobacteriaceae* were quantified on Violet Red Bile Agar (VRBG) by pour plating. Plates were incubated at 37 °C for 24 h [29]. Presumptive *Escherichia coli* was enumerated by an additional set of VRBG Agar plates incubated at 44 °C for 24 h [30,31].

(c) Enumeration of *Bacillus cereus*, *Bacillus* spp., *Staphylococcus aureus* and *Escherichia coli*

To enumerate *B. cereus* and other aerobic spore formers, the serial dilution tubes were heated for 13 min. in a water bath at 80 °C before plating. *B. cereus* was enumerated by surface plating on Mannitol Egg-yolk Polymyxin Agar (MYPA) followed by incubation at 37 °C for 24 h. All large, rounded colonies, pink in color and surrounded by a precipitation zone were enumerated as presumptive *B. cereus* [32,33].

*Bacillus* spp. counts were quantified on Reinforced Clostridial Agar incubated aerobically at 37 °C for 24 h.

*S. aureus* was quantified on Baird-Parker Agar with egg-yolk tellurite by surface plating followed by incubation at 37 °C for 48 h. Gray-black colonies with haloes were enumerated as presumptive *S. aureus* [34,35].

Presumptive colonies of *E. coli, B. cereus* and *S. aureus* were confirmed using PCR as described by [36]. Microbial counts were expressed as log10 mean (*n* = 3) cfu/g of cassava solid sample or cfu/mL of cassava press water sample.

All microbial analyses described above were performed for samples collected within the whole chain of cassava processing at *Unit J*, whereas for “rale” (the finished product) collected both in different markets and different cassava processing units, we focused on enumeration of yeasts, molds, *S. aureus*, TBC, *Enterobacteriaceae* and *B. cereus*.

### 2.4. Yeast and Mold Identification

Preliminary identifications of purified representative isolates as described in Section 2.3–a), were based on macro and micro-morphology. Isolates that appeared to have similar macro and micro-morphology were grouped and given codes for further steps. These representative yeast and mold isolates were grown in Yeast Extract Peptone Dextrose broth (20 g/L peptone, 10 g/L Yeast extract, 20 g/L glucose) for DNA extraction and sequence-based species identification. 

PCR analysis was performed for yeast isolates by selecting single colonies as templates. Material from a single yeast colony was boiled in 20 μL 0.02 M NaOH for 5 min, and 2 μL of the resulting suspension was used as PCR template with primers NL1/NL4 to amplify the D1/D2 region of the 26S rRNA gene [37,38]. For molds, DNA was extracted using the method described by [39]. The following genes were selected for identification by partial amplification and sequencing: translation elongation factor 1 α for presumptive *Fusarium* spp. with primers EF1/EF2 [40]; β-tubulin gene in *Penicillium* subgenus *Penicillium* spp. with primers bt2a/bt2b [41]; and rDNA internal transcribed spacer in all other species with primers ITS1F/ITS4 [42]. Amplicons were sequenced at Macrogen, Amsterdam, The Netherlands, and all representative isolates were identified by sequence search and comparison against the NCBI database (Nucleotide Blast, Core nucleotide database “https://blast.ncbi.nlm.nih.gov/Blast.cgi?PROGRAM=blastn&PAGE_TYPE=BlaSearch&LINK_LOC=blasthome (accessed on 25 June 2023)”.

### 2.5. Culture-Independent Analysis of Bacterial Community by Illumina Amplicon Sequencing

16S rRNA gene sequencing was applied as a culture-independent method to gain an overview of bacterial diversity. DNA was extracted using a Quick-DNA Fungal/Bacterial Microprep Kit (Zymo Research™, Freiburg, Germany) including an additional bead-beating step: briefly, 20 ± 10 mg sample was weighed in ZR BashingBead™ Lysis tubes (Freiburg, Germany) and placed on ice. A total of 750 μL of BashingBead™ Buffer (Freiburg, Germany) was added in the sample directly to the tube and capped tightly. The tube was placed in a FastPrep Instrument (MPBiomedicals™, Freiburg, Germany) for 40 s at speed setting 6.0 and centrifuged at 10,000× *g* for 10 min at room temperature (20 °C). Then, 400 μL of the obtained supernatant was transferred into a new microcentrifuge tube and treated as described in the manufacturer’s protocol. The DNA concentration was approximated using Qubit© fluorometer dsDNA protocol (Invitrogen –Thermo Fisher Scientific, Dreieich, Germany). DNA was purified in triplicates for all samples, and some replicates had to be diluted before PCR to overcome inhibitory effects. PCR amplification, purification, and barcoding/preparation of libraries for Illumina Sequencing were performed using the protocol modified by [43].

16S rRNA gene amplicon libraries were constructed as triplicates using two consecutive PCR procedures. The first PCR targeted and amplified the V4 region of bacteria, using the primers 515F (ACACTCTTTCCCTACACGACGCTCTTCCGATCTNNNNGTGBCAGCMGCCGCGAA) and 805R (AGACGTGTGCTCTTCCGATCTGGACTACHVGGGTWTCTAAT), and it attaches adaptors to the amplicons [43]. The reaction mixture contained 2× Phusion High-Fidelity DNA Polymerase/dNTP mix (Thermo Fischer Scientific, Hudson, NH, USA), 10 μM of each primer, and approx. 5–10 ng DNA template in a final volume of 25 μL. The condition for amplification was as follows: initial denaturing at 98 °C for 30 s, 30 cycles of 10 s at 98 °C, 30 s at 60 °C, 4 s at 72 °C, and a final extension at 72 °C for 2 min. The PCR products were checked for size and quality by electrophoresis.

Amplicons were purified using Agencourt AMPure XP (Becker Coulter, Brea, CA, USA), using a magnetic particle/DNA volume ratio of 0.8:1. In the second PCR, Illumina-compatible barcodes were added to the amplicons [44]. The PCR reaction contained 10 μL purified amplicon from the first step, 2× Phusion High-Fidelity DNA Polymerase/dNTP mix and 10 μM each of the primers 5’-AATGATACGGCGACCACCAGATCTACACX8ACACTCTTTCCCTACACGACG-3’ and 5′-CAAGCAGAAGACGGCATACGAGATX8GTGACTGGAGTTCAGACGTGTGCTCTTCCGATCT-3′, where X8 in the primer sequence represented a specific Illumina-compatible barcode (Eurofins– Genomics). The total volume was 25 μL. The following conditions were used for the second PCR step: initial denaturing at 98 °C for 30 s, 8 cycles of 10 s at 98 °C, 30 s at 62 °C, 5 s at 72 °C, and a final extension at 72 °C for 2 min. The PCR products were checked by electrophoresis and purified using Agencourt AMPure XP. The PCR products were then each diluted to a DNA concentration of approx. 24 nM and pooled together.

Pair-end sequencing was performed on the MiSeq platform (Illumina, Inc., San Diego, CA, USA) at ScilifeLab, National Genomics Infrastructure (Stockholm, Sweden). Amplicon sequence variants, abundancies and taxonomic affiliation were determined using the package dada2 (version 1.6.0) [45] in R (version 3.4.0), which is implemented on the SLUBI computing cluster in Uppsala (running on CentOS Linux release 7.1.1503; module handling by Modules based on Lua: Version 6.0.1 “https://www.slubi.se/ (accessed on 16 October 2023)”. For further details see [44].

### 2.6. Statistical Analysis

Statistical analysis was carried out using R in the RStudio (2024.09.1) environment [46]. The normality function from the *dlookr* version 0.6.3 package [47] was used to retrieve the results of the *Shapiro* test and therefore assess whether the collected data fulfil the assumptions for the ANOVA test. The *Bartlett’s* test was performed using an inbuilt function from the R software (version 4.3.2) to assess other ANOVA assumptions (homogeneity of variances). Since the assumptions were not satisfied, the *Kruskal–Wallis’s* test was used as a non-parametric alternative to ANOVA. The *Dunn* test was performed as a post-hoc test to derive pairwise multiple comparisons among significant groups of microorganisms in *Cassava Processing Stages*, *Cassava Processing Units* and *Rural Markets*. The *p*-values for multiple comparisons were adjusted using the Bonferroni method. All results were considered significant at *p* < 0.05. In general, the *gtsummary* package version 1.7.2 [48] was used to compute both the descriptive statistics and inferential statistics. The *rstatix* package version 0.7.2 [49] was used to derive both the *Kruskal–Wallis’s* and *Dunn* tests.

For Illumina 16S rRNA gene sequencing results, all abundances below the cut-off value of 0.5% were removed from considerations.

## 3. Results

### 3.1. Microbes as Indicators of Hygienic Quality Within Unit J

Table 1 describes microbes indicative of hygienic quality isolated from cassava samples collected in different stages of cassava processing within *Unit J* during the rainy and dry seasons. Molds, Lactic Acid Bacteria (LAB), Aerobic bacteria (Total Aerobic Bacteria Counts, TBC) and *Bacillus* spp. were observed in the cassava samples from the rainy season, and the presence of yeasts and *S. aureus* were found in cassava samples collected during the dry season.

The counts of microorganisms in the processing chain of cassava differ significantly between each stage of processing cassava tubers to cassava roasted flour (“rale”) in both rainy and dry seasons, as well as when combining all seasons (Table 1). The dry season generally reported higher counts of microorganisms in the cassava processing chain compared to the rainy season. On both occasions, the counts of *Enterobacteriaceae*, presence of *E. coli*, and *B. cereus* were below the detection limit (Table 1).

Specifically, in the rainy season, the levels of contamination of cassava samples by yeasts and *S. aureus* were below the detection limit in all processing stages. Mold counts were generally low, with the roasting process having the highest counts (mean 1.33 log cfu/g) followed by chopping and pressing stages (mean 0.33 log cfu/g). LAB, TBC and *Bacillus* spp. were only detected during the chopping, pressing, and sieving stages, with counts ranging from 5.54 to 6.81 log cfu/g for LAB, 5.32 to 6.53 log cfu/g for TBC, and 3.04 to 3.33 log cfu/g for *Bacillus* spp. (Table 1).

In contrast, during the dry season, the presence of yeasts and *S. aureus* were ob-served in the stages of chopping, pressing, and sieving. The pressing stage recorded the highest cfu in both groups of microorganisms (4.21 log cfu/g for yeasts and 5.73 log cfu/g for *S. aureus*, respectively). In this season, molds, LAB, and TBC were also present during all cassava processing stages. The highest mold count was observed in the roasting process (2.72 log cfu/g), while the lowest values were reported during the sieving stage (*bdl*). For LAB and TBC, the highest cfu counts were observed in the pressing stage (6.50 log cfu/g for LAB and 6.22 log cfu/g for TBC), and the lowest values were found for the press water collected during the pressing stage (*bdl* for LAB and 2.23 log cfu/mL for TBC). The presence of *Bacillus* spp. in this season was below the limit of detection (Table 1). When combining all seasons of study, significant differences were observed within cassava processing stages for LAB, TBC and *Bacillus* spp.

In the rainy season, molds of the genus *Penicillium* (*P. ochrochloron*, *P. primulinum*, *P. citreonigrum*) were isolated from chopping, pressing and press water samples, whereas the roasting stage was more contaminated by *Alternaria infectoria*, *Cladosporium sphaerospermum* and *A. flavus*. No yeast contamination was observed in all cassava samples assessed in this season (Table 2). 

The contamination of the cassava samples by both molds and yeasts was higher during the dry season than the rainy season. Samples were contaminated by *Aspergillus fumigatus*, *Penicillium griseofulvum* and *Rhizopus stolonifer* at the pressing stage, and *Cladosporium cladosporioides*, *Cladosporium oxysporum* and *R. stolonifer* at the roasting stage. In contrast, the chopping, pressing and sieving stages were found to have the highest diversity of yeasts. *Wickerhamomyces anomalus*, *Rhodotorula mucilaginosa*, *Pichia exigua* and *Meyerozyma caribbica* were the most frequent yeast species found in these cassava processing stages. *Rhodotorula babjevae*, a red oleaginous yeast, was also observed in the press water samples. 

All samples from the sieving stage were free from contamination by molds in both surveyed seasons (Table 2). Certain microbial isolates could not be identified in the present study due to the lack of reference sequences in the NCBI database (Nucleotide Blast, Core nucleotide database) “https://blast.ncbi.nlm.nih.gov/Blast.cgi?PROGRAM=blastn&PAGE_TYPE=BlastSearch&LINK_LOC=blasthome (accessed on 15 January 2022)”. However, identified isolates presented here were deemed sufficient to give an indication of the types of species present (Table 2).

**Figure 2:**
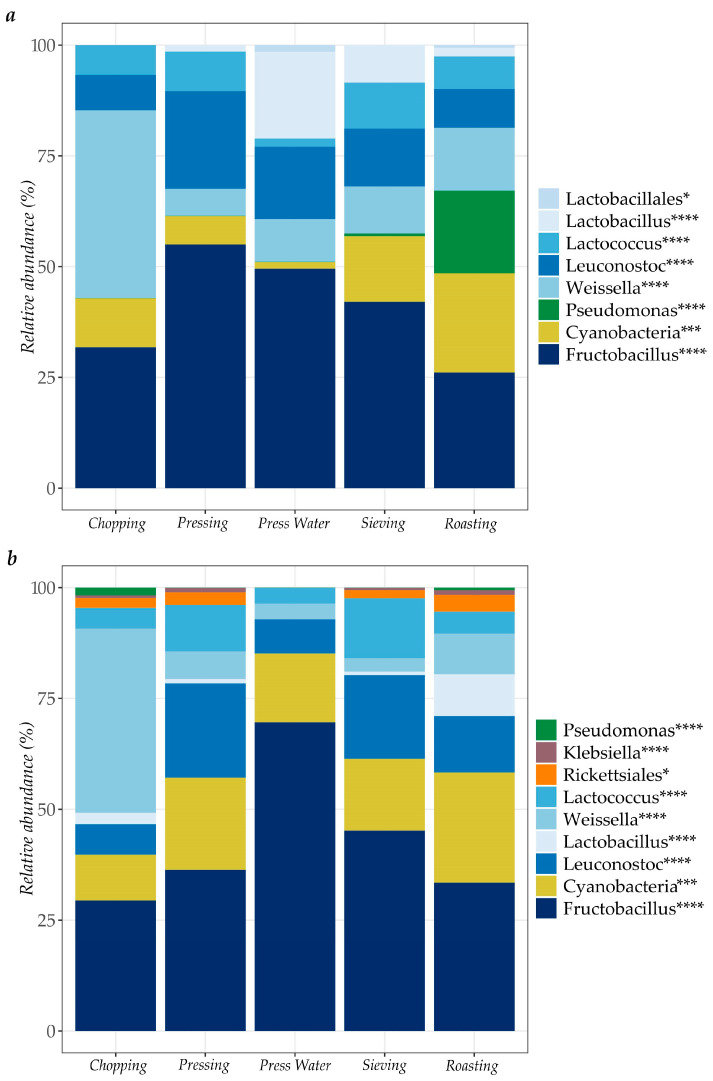
Relative abundance of bacteria at order *, class ***, and genus **** levels in cassava samples (*n* = 3) collected in different stages of processing within *Unit J* during: (**a**) the rainy season (November 2020) and (**b**) the dry season (August 2021).

Illumina 16S rRNA gene sequencing analysis of the bacterial microbiota revealed high prevalence of LAB and Lactobacillales in all samples for the two seasons of the study. The LAB affiliates to the genera *Fructobacillus*, *Lactobacillus*, *Lactococcus*, *Leuconostoc* and *Weissella* (Figure 2). This included samples from the roasting stage during the rainy season where LAB counts were below the detection limit (Table 1). Amplicons representing Cyanobacteria were also abundantly found in the samples. Regarding Gram-negative bacteria, in both seasons, bacteria from the order Rickettsiales, and the genera *Pseudomonas* and *Klebsiella* were observed at somewhat greater abundance in samples collected at the roasting stage. Bacterial species of *Aeromonas* and *Escherichia*/*Shigella* were also found in samples collected in the roasting stage within the first sampling period.

### 3.2. Microbes as Indicators of Hygienic Quality in “Rale” Sampled from Cassava Processing Units

Counts of microorganisms from the final cassava product (“rale”) differ significantly between the six main cassava processing units located in Gaza and Inhambane provinces, Mozambique, in both the rainy and dry seasons (Table 3). However, no significant difference in terms of microbial contamination was found when combining the two seasons. On all occasions, yeasts and *Enterobacteriaceae* were always found to be below the limit of detection.

During the rainy season, molds appeared to dominate most samples collected from all cassava processing units, with counts varying from 0.20 log cfu/g (*Unit W*) to 3.47 log cfu/g (*Unit Z*). TBC could only be quantified in samples collected in *Unit C* (3.33 log cfu/g), *Unit J* (6.67 log cfu/g) and *Unit V* (7.30 log cfu/g), while *S. aureus* was only found in *Unit W* (2.33 log cfu/g). *B. cereus* was not detected in any of the samples. 

In the dry season, TBCs were more frequently isolated than other groups of tested microorganisms, varying from 1.34 log cfu/g (*Unit J*) to 1.83 log cfu/g (*Unit V*). Molds were observed in four out of the six associations included in this study, ranging from 0.89 log cfu/g (*Unit C*) to 3.90 log cfu/g (*Unit V*). The highest counts of *B. cereus* were reported at *Unit C* (2.33 log cfu/g). Similar to the rainy season, *S. aureus* was reported only at *Unit W* (2 log cfu/g), while in other units the counts for this microbe were below the level of detection (Table 3).

The highest diversity of molds in “rale” samples collected at the six cassava processing units was observed in the dry season (Table 4). *Penicillium* spp., *Aspergillus* spp., *Fusarium* spp., and *Alternaria* spp. were reported in both seasons.

**Figure 3:**
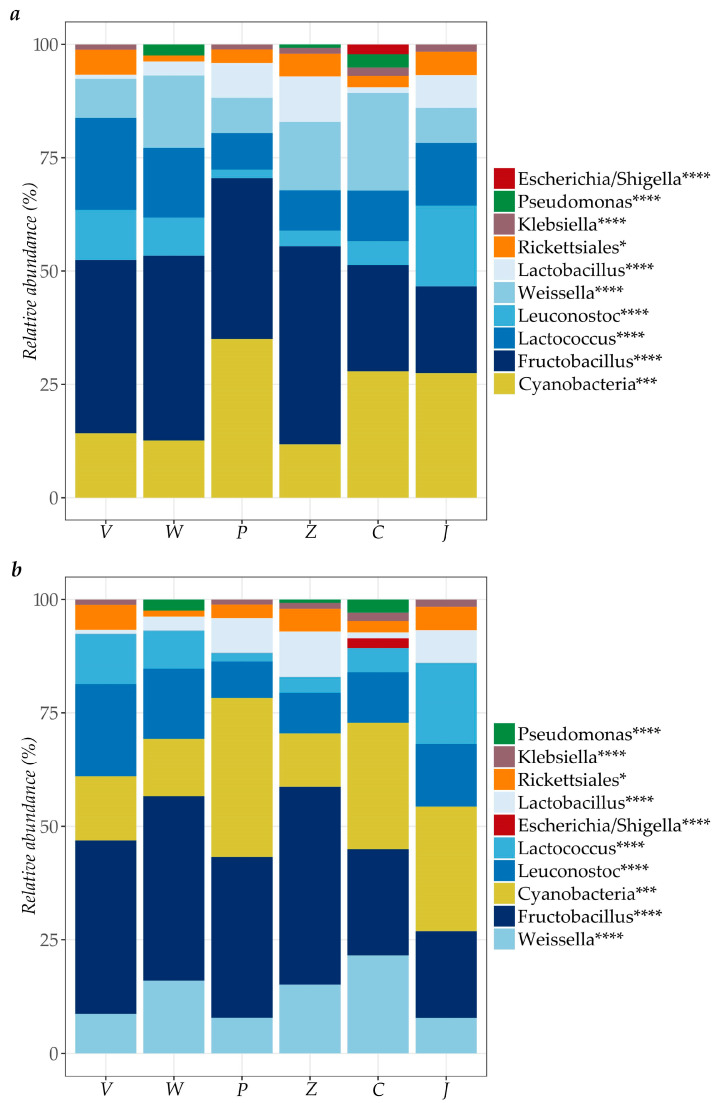
Relative abundance of bacteria at order *, class ***, and genus **** levels in ”rale” samples (*n* = 3) collected in six different cassava processing units during (**a**) the rainy season (November 2020) and (**b**) the dry season (August 2021).

Relative abundance of bacterial groups in “rale” samples collected in six different cassava processing units is shown in Figure 3. LAB (*Fructobacillus*, *Lactobacillus*, *Lactococcus*, *Leuconostoc* and *Weisiella*) and the Cyanobacteria class were the most dominant in samples collected both in dry and rainy seasons, and in all cassava processing units. Other genera present included *Klebsiella*, *Escherichia/Shigella*, *Cloacibacterium* and *Staphylococcus*, as well as the order Rickettsiales and family *Neisseriaceae*.

The Gram-negative genera *Pseudoxanthomonas* and *Pseudomonas* were also occasionally present in “rale” samples, and *Unit Z* was found to have the highest diversity of bacterial microbiota.

### 3.3. Microbes as Indicators of Hygienic Quality of “Rale” Collected in Rural Markets

Table 5 describes the enumeration of various microbes in “rale” samples collected in five rural markets, where “rale” is traditionally sold. The diversity of microorganisms varied between the two seasons of study within the five assessed markets. No significant differences in microbial diversity were found between the rural markets during the rainy season, whereas significant differences were found during the dry season and when combining the microbe counts from both seasons.

In the rainy season, *S. aureus* was only confirmed from one market (*MK*) at 2.01 log cfu/g. The same scenario was observed for TBC, which were only reported in one market (*GB*) at 2.05 log cfu/g. The counts for yeasts, *Enterobacteriaceae* and *B. cereus* were below the detection level. Average mold counts were very low, as reflected by a few colonies on occasional plates (Table 5).

In contrast, in the dry season, the highest counts for yeasts (4.55 log cfu/g), molds (2.32 log cfu/g), and *S. aureus* (2.16 log cfu/g) were observed in samples belonging to the *MC* market. All market samples were positive for TBC, with values ranging from 1.71 log cfu/g (*MK*) to 2.54 log cfu/g (*GB*). Market *MC* had the highest counts for *B. cereus* (2.33 log cfu/g) followed by market *AM* (2.01 log cfu/g). In this season, all market samples were found to be negative for *Enterobacteriaceae*. When combining both seasons of study, significant differences of microbial contamination were observed among rural markets for molds and TBC (Table 5).

Molds identified in “rale” samples collected in rural markets during the rainy season belonged to the genera *Fusarium*, *Rhizopus* and *Talaromyces* (Table 6). In the dry season, *Pithomyces*, *Aspergillus*, *Talaromyces*, and *Trematosphaeia* were identified. The *MK* market samples had somewhat higher mold counts and much greater species diversity during the dry season compared to the rainy season.

**Figure 4:**
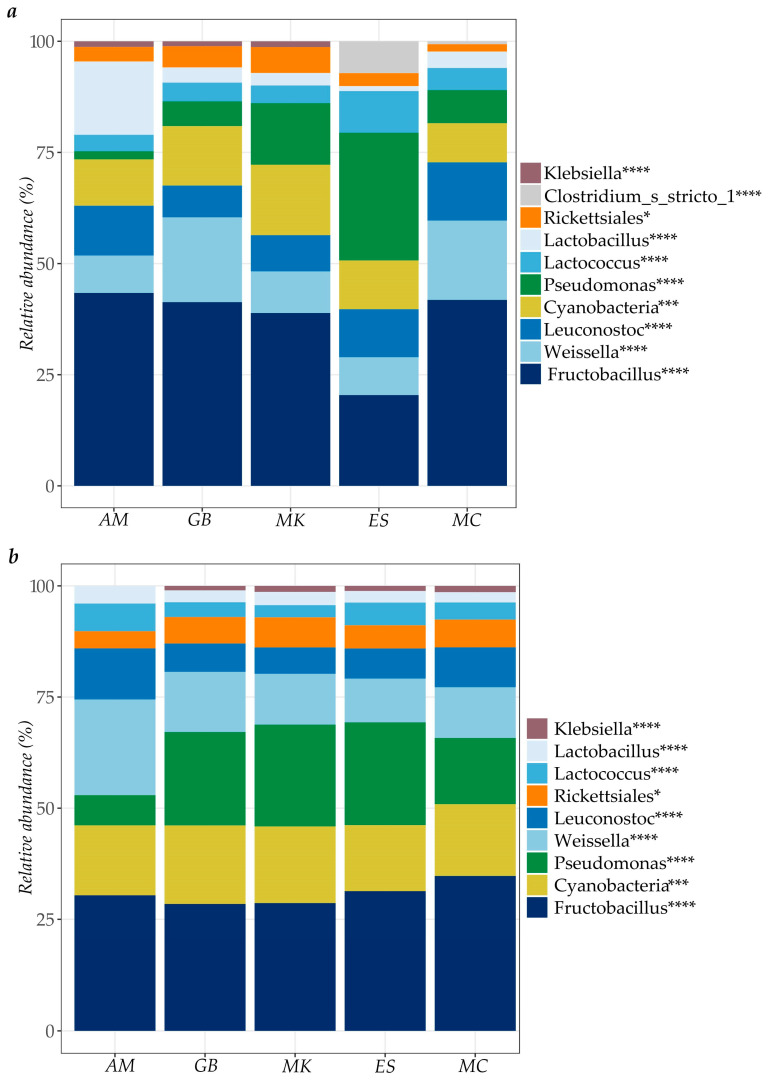
Relative abundance of bacteria at order *, class ***, and genus **** levels in “rale” samples (*n* = 3) collected in five different rural markets during (**a**) the rainy season (November 2020) and (**b**) the dry season (August 2021).

The composition of the bacterial community displayed as relative abundance from “rale” samples collected in rural markets are presented in Figure 4. During both seasons, bacteria from the class Cyanobacteria, and the genera *Fructobacillus*, *Lactobacillus, Lactococcus*, *Leuconostoc* and the *Weisiella* genus were most frequent in all surveyed markets. Regarding the Gram-negative community, the genus *Klebsiella* and *Acinetobacter*, the family *Neisseriaceae*, and the order Rickettsiales were also dominant in samples from both seasons. The samples from markets *AM* and *ES* reported the highest relative abundance of bacterial community within the rainy season. In contrast, during the dry season, “rale” sold in the *GB*, *MK* and *MC* markets showed high prevalence of bacterial populations, complementing the results found with the plating method (Table 5).

## 4. Discussion

### 4.1. Microbes as Indicators of Hygienic Quality Within Unit J

During the processing chain to produce cassava roasted flour (“rale”), the highest counts of molds and bacteria were found precisely after chopping. This can be explained by the high level of humidity that is found in cassava roots [50]. Cassava roots consist of, on average, 70% moisture, which requires prompt processing after harvesting to increase the shelf life of the cassava root products [22]. The rainy season is the most challenging season for Mozambican cassava farmers, as the processing and storage of cassava and its derivatives are more susceptible to mold contamination due to high temperatures and relative humidity (± 29 °C ± 75% HR) at the locations of the associations included in the study. High TBCs were reported by [51] in “rale” samples and they correlated their findings with the contamination of samples by bacteria both from the cassava processors (when handling the flour) and from the environment.

*S. aureus* was isolated during the dry season at various stages of cassava processing (Table 1). According to [52], the presence of *S. aureus* in processed foods, or on food processing equipment, is generally an indication of inadequate sanitation or handling [52]. There are many records of severe food poisoning outbreaks caused by this microorganism. *S. aureus* can contaminate food processes when handling with bare hands; their presence in cassava samples might be related to direct contact or air-droplet mechanisms such as coughing or sneezing by “rale” processors. Foods contaminated by *S. aureus*, *Bacillus* spp., *Shigella* sp., and *Enterobacter* sp. have been connected to food infections and intoxication leading to different forms of diarrhea diseases among other complications, especially in young children, the elderly and the immunocompromised [51,53,54]. Fortunately, the production of “rale” includes heat treatment during roasting at approx. 110 °C, which is sufficient to eliminate these microbes (as well as natural microbiota such as LAB), though spores of *Bacillus* sp. may survive. In this context, it is noteworthy that *S. aureus* in certain samples from the dry season (Table 1) approaches levels of >10^6^ cfu/g which indicate a risk for the production of a heat-stable toxin which would still be present in the roasted product [54,55].

Recontamination of samples after roasting may occur if ideal storage conditions are not put in place [51]. As the water content is reduced during roasting, this favors in particular the presence of molds (Table 1), which thrive at low water activity. Additionally, the roasting process in the rainy season samples took place outdoors using less advanced roasters, while for the subsequent dry season, a new roasting machine had been installed in *Unit J*. Rainy season samples were at greater risk for contamination by soil residues, the surrounding environment and sweat, as well as the lack of awareness of hygiene practices when carrying out this activity.

Various mold species were isolated from cassava processing samples (Table 2), including *Penicillium* and *Aspergillus* species, which have been previously reported from cassava derivatives in Benin [56] and Ghana [57]. Some of these molds may cause physical–chemical damage in the product and even potentially affect human and animal health, via the production of aflatoxins and other mycotoxins [58,59,60,61]. Fifteen samples of cassava flour in Brazil were investigated by [62] and this study concluded that 80% of the samples were contaminated by *A. niger*, *A. fumigatus* and *Penicillium* species. Contamination of cassava samples by *Penicillium* spp., *Aspergillus* spp., genera *Rhizopus* and *Cladosporium*, and yeasts were reported by [63] in Brazil. In our study, only low colony counts of potentially toxigenic molds such as *A. flavus* were detected in ”rale” samples. This would seem to indicate that the risk for aflatoxin contamination is fairly low [64] in the “rale” produced and processed at *Unit J* using the current technology. Likewise, although citreoviridin may have hypothetically been produced by *P. citreonigrum*, the counts were low (Table 2) and the occurrence of this toxin in foods is not regulated, despite its past role in causing *beriberi* from contaminated rice [65]. A pre-study we conducted in February 2020 in Gaza and Inhambane provinces showed similar trends regarding yeasts, molds, LAB, *Enterobacteriaceae*, TBC and *Bacillus* spp. in samples from the cassava processing chain, suggesting that the results from subsequent seasons (November 2020 and August 2021) were not unusual. *Penicillium* sp., *Aspergillus* sp., *Aureobasidium* sp. and *Cladosporium* sp. were also isolated in the pre-study.

### 4.2. Microbes as Indicators of Hygienic Quality in “Rale” Sampled from Cassava Processing Units

Regarding the study performed in the cassava processing units, our findings show concordance with the work carried out by [66,67] in Brazil, where they observed high records of mold in cassava flour in at least 67 to 75% of the samples. Studies carried out by [68] in Nigeria reported mold counts ranging from 3.55 to 5.99 log cfu/g, which represents higher contaminations of samples compared to the units of the current study (Table 3). The maximum allowable level for molds in cassava flour is 10^3^ cfu/g or 3 log cfu/g [64], meaning that the counts observed in the “rale” processed in the majority of units meet the limits; this is probably assisted by the roasting process which reduces the initial mold load. However, *Units V* and *Z* were slightly over the limit. These units are smaller, processing less volumes than the other units, and therefore, the sampled “rale” was either not fresh or properly stored, contributing to the very high microbial counts.

*B. cereus* was isolated at low levels from a few units. This can be related to the occurrence of this bacteria in soil, and, from there, its contamination of cereals, tubers and vegetables [69]. *S. aureus* was reported in the most rudimentary unit (*Unit W*) that relies on very old machines and less advanced infrastructure to produce, process and store cassava derivatives. Consequently, our study strongly suggests that reducing contamination from handling is a challenge when using old machinery and infrastructure. The contamination of samples by both molds and *E. coli* reduces the conformity of the cassava product to microbial quality and safety regulations [64].

The microbes identified in the present study are aligned with those obtained by [68], in which they reported the presence of *Aspergillus* spp., *Penicillium* spp., *Fusarium*, *Alternaria* spp., *Cladosporium* sp. and *Rhizopus* sp. in cassava roasted flour samples. Most species listed in Table 2 are non-toxigenic, however, the list does include *A. flavus*, a producer of aflatoxin; *P. citrinum*, a producer of citrinin; *A. niger*, producer of ochratoxin A and fumonisins; and *F. oxysporum*, where a few strains produce fumonisin [65,70]. These toxins are among those whose occurrence in certain foods is regulated in the European Union [71]. Other species producing non-regulated mycotoxins include *P. citreonigrum* (citreoviridin, discussed in Section 4.1) and *A. alternata* (alternariols, tenuazonic acid) [65]. Despite the presence of these species, the risk for mycotoxin production is deemed to be low, because the mold counts were fairly low in all samples (<10^4^ cfu/g or 4 log cfu/g), and in particular, *A. flavus* was present at <3 cfu/g or 0.5 log cfu/g. The toxigenic molds are only likely to pose a risk if the “rale” is stored for long periods with high humidity, which would permit mold growth and toxin production.

### 4.3. Microbes as Indicators of Hygienic Quality in “Rale” Collected in Rural Markets

In general, the market samples revealed lower counts and less diversity of microorganisms compared to the cassava processing unit samples. Furthermore, mycotoxigenic mold species were not observed in the market samples. The presence of molds, yeasts and *S. aureus* in certain market samples might be related with variations in personal hygiene and food safety consciousness, e.g., using clean containers with covers to store the “rale” that is sold in markets as a way to reduce the direct and indirect contaminations by air-borne droplets and molds and yeast in dust, soil, and air [51]. High loads of bacteria, *Staphylococcus* spp. and coliforms were reported by [72] in dried chips and cassava flour samples in Kenya. These findings were correlated with excessive personnel handling and insufficient hygiene applied during post-harvest processing, handling, and marketing. The same study confirmed the occurrence of high mold counts in cassava flour because of storage practices of the products, meaning that it is very difficult to follow the hygiene-safety regulations outside the processing unit.

The “rale” sold in rural markets of Gaza and Inhambane is usually home-made or mainly produced in artisanal processing units that do not rely on proper processing equipment/technology. This tradition of processing cassava hypothetically contributes to fluctuating hygienic quality in the final product. The goal of standardizing small–scale processing products is a challenge. For instance, in their review, the authors [15] mention that the desirable attributes of cassava flour differ across ethnicities and regions, and emphasize that varying quality of the products among processors and even between batches from the same processor hinders commercialization of locally produced cassava products.

The risk for contamination of “rale” sold in rural markets by molds can be associated with lack of awareness and improper handling by the vendors. Open containers used by vendors to stock the cassava flour in the markets means that they are constantly exposed to air, which permits mold spores and other microbes to contaminate the product [51]. Proper training on good practices especially good hygiene, as well as equipping farmers, processors and retailers with more hygienic equipment and methods, could be a strategy to reduce microbial loads on the cassava products available in the market, leading to improved quality and safety [72,73].

### 4.4. Culture-Independent Analysis of Bacterial Community

LAB were the dominant microbes (present at >50%) found at all cassava processing stages and in the final “rale” product collected from all of the included processing units (Figure 2, Figure 3 and Figure 4). These are the primary fermentation organisms during cassava wet-processing and therefore key members of cassava natural microbiota [74]. This is reflected in the relatively high abundance of amplicons from DNA of lactic acid bacteria during processing and in all subsequent samples taken thereafter. The sequencing method applied in this study does not distinguish between DNA from live and dead bacteria, meaning that some of the DNA could be carried over from bacteria present in earlier stages of processing but which are non-viable.

*Enterobacteriaceae* (*Klebsiella*, *E. coli*/*Shigella*) were detected at low relative abundance in many samples during processing (<2% relative abundance, Figure 2), in “rale” collected from six units (<15% relative abundance, Figure 3) and in “rale” from markets (<5% relative abundance, Figure 4). These genera are indicators of poor hygiene linked to fecal contamination of, for example, processing water or from handlers [75]. However, *Enterobacteriaceae* were not detected during plating of any samples (detection limit < 33 cfu/g or 1.52 log cfu/g). Despite some potential loss of viability among Gram-negative bacteria during freeze-storage of the samples, the overall picture is that this group was unlikely to be present at levels posing a health risk.

*Pseudomonas*, a commonly abundant environmental and spoilage bacteria [76], was found to be present at low relative abundance during processing (<20% relative abundance, Figure 2) and in “rale” collected from six processing units (<5% relative abundance, Figure 3), but it was increased in abundance in “rale” samples collected at rural markets (50–80% relative abundance, Figure 4). This increase is due to aerobic storage and handling of the “rale” prior to being sold at the markets.

Thus, the results obtained from the Illumina 16S rRNA gene sequencing are in accordance with the trends observed by the plating method, and can be considered a complementary technique to the plating methods applied in this and previous studies e.g., [51,64].

## 5. Conclusions

This study revealed differences in terms of the microbial contamination within the processing chain of cassava roots to cassava roasted flour produced at processing *Unit J* in the surveyed seasons. Lack of hygiene practices during the processing chain of cassava by the processors might lead to high levels of microbial contamination, compromising the final quality of the product and the food safety for the consumers. Hence, it is important to maintain high quality and safety process standards in place at *Unit J*.

High mold counts in cassava roasted flour indicates anomalies during storage of the cassava product “rale”, at the production facilities and/or the markets. The low counts of microorganisms found in “rale” collected both in cassava processing units as well as in the rural markets suggests an acceptable quality of the product for human consumption. To acquire a better overview of microbial hygiene within the markets, frequent sampling would be necessary. However, our study gives indications that the hygiene is reasonable during both the dry and rainy seasons.

The results obtained within this study did not point towards any risks for aflatoxin contamination in cassava samples. The inclusion of more processing units that rely on rudimentary equipment for processing cassava would broaden our understanding of possible variations in microbiota during cassava processing. However, it is still important to maintain and reinforce basic hygiene and sanitary practices to improve quality and safety of the cassava derivatives in southern Mozambique.

## Figures and Tables

**Table 1:** Enumeration (log cfu/g) of different microbial groups isolated within *Unit J* during rainy, dry and both seasons. Values are presented as mean ± standard deviation (*n* = 3 for each season and *n* = 6 for both seasons). Different superscript letters represent significant differences (*p* < 0.05).

Cassava Processing Stages						
Microbes	*Chopping ^1^*	*Pressing ^1^*	*Press Water ^1^*	*Sieving ^1^*	*Roasting ^1^*	*p-Value ^2^*
**Rainy season (November 2020)**						
Yeast	*bdl*	*bdl*	*bdl*	*bdl*	*bdl*	*na*
Molds	0.33 ± 0.58	0.33 ± 0.58	0.10 ± 0.17	▪	1.33 ± 1.59	0.52
LAB	5.54 ± 0.13 ^a^	6.81 ± 0.90 ^a^	*bdl*	5.71 ± 0.34 ^a^	*bdl*	0.011 *
*S. aureus*	*bdl*	*bdl*	*bdl*	*bdl*	*bdl*	*na*
TBC	5.32 ± 0.08 ^a^	6.17 ± 0.10 ^a^	*bdl*	6.53 ± 0.4 ^a^	*bdl*	0.009 **
*Enterobacteriaceae*	*bdl*	*bdl*	*bdl*	*bdl*	*bdl*	*na*
Pres. of *E. coli*	*bdl*	*bdl*	*bdl*	*bdl*	*bdl*	*na*
*Bacillus* spp.	3.04 ± 0.87 ^a^	3.33 ± 1.15 ^a^	*bdl*	3.05 ± 0.40 ^a^	*bdl*	0.026 *
*Bacillus cereus*	*bdl*	*bdl*	*bdl*	*bdl*	*bdl*	*na*
**Dry season (August 2021)**						
Yeast	4.13 ± 0.34 ^a^	4.21 ± 0.05 ^a^	2.84 ± 0.79 ^a^	4.02 ± 0.18 ^a^	*bdl*	0.026 *
Molds	0.67 ± 1.15	0.40 ± 0.17	0.58 ± 0.51	*bdl*	2.72 ± 0.13	0.05
LAB	4.52 ± 0.31 ^a^	6.50 ± 0.65 ^a^	*bdl*	*bdl*	3.35 ± 0.60 ^a^	0.011 *
*S. aureus*	5.61 ± 0.12 ^a^	5.73 ± 0.05 ^a^	*bdl*	5.50 ± 0.56 ^a^	*bdl*	0.024 *
TBC	5.90 ± 0.21 ^ab^	6.22 ± 0.27 ^a^	2.23 ± 0.11 ^b^	5.67 ± 0.30 ^ab^	3.25 ± 1.08 ^ab^	0.017 *
*Enterobacteriaceae*	*bdl*	*bdl*	*bdl*	*bdl*	*bdl*	*na*
Pres. of *E. coli*	*bdl*	*bdl*	*bdl*	*bdl*	*bdl*	*na*
*Bacillus* spp.	3.04 ± 0.87 ^a^	3.33 ± 1.15 ^a^	*bdl*	3.05 ± 0.40 ^a^	*bdl*	0.028 *
*Bacillus cereus*	*bdl*	*bdl*	*bdl*	*bdl*	*bdl*	*na*
**All seasons (Rainy and Dry)**						
Yeast	2.91 ± 1.35	2.96 ± 1.38	2.27 ± 0.80	2.86 ± 1.28	1.85 ± 0.16	0.81
Molds	0.33 ± 0.82	0.20 ± 0.25	1.41 ± 1.44	*bdl*	0.37 ± 0.43	0.1
LAB	5.03 ± 0.60 ^ab^	6.65 ± 0.72 ^a^	2.36 ± 0.40 ^b^	6.10 ± 0.47 ^a^	2.67 ± 0.83 ^b^	<0.001 ***
*S. aureus*	3.80 ± 1.98	3.87 ± 2.04	*bdl*	3.75 ± 1.95	*bdl*	0.09
TBC	5.61 ± 0.35 ^ab^	6.19 ± 0.18 ^a^	2.11 ± 0.14 ^b^	6.10 ± 0.57 ^a^	2.62 ± 0.97 ^b^	<0.001 ***
*Enterobacteriaceae*	*bdl*	*bdl*	*bdl*	*bdl*	*bdl*	*na*
Pres. of *E. coli*	*bdl*	*bdl*	*bdl*	*bdl*	*bdl*	*na*
*Bacillus* spp.	3.04 ± 0.78 ^a^	3.33 ± 1.03 ^a^	1.85 ± 0.16 ^b^	3.05 ± 0.36 ^a^	*bdl*	<0.001 ***
*Bacillus cereus*	*bdl*	*bdl*	*bdl*	*bdl*	*bdl*	*na*

Abbreviations: *bdl*—below the detection limit; *na*—not applicable; LAB—lactic acid bacteria; TBC—total bacterial count; ▪—less than two colonies/plate (10^−1^); cfu— colony forming units; ^1^ Mean (SD); ^2^* *p* < 0.05; ** *p* < 0.01; *** *p* < 0.001.

**Table 2:** Molds and yeasts (% of isolates) identified in cassava samples from *Unit J* during rainy and dry seasons.

	Rainy Season (November 2020)			Dry Season (August 2021)	
	Mold Isolates	Yeast Isolates		Mold Isolates	Yeast Isolates
*Chopping*	*Penicillium ochrochloron* (100%)	-		*Curvularia* sp. (50%)	*Wickerhamomyces anomalus* (71%)
				*Rhizopus stolonifer* (50%)	*Rhodotorula mucilaginosa* (14%)
					*Pichia exigua* (7.5%)
					*Rhodotorula alborubescens* (7.5%)
*Pressing*	*Penicillium primulinum* (50%)	*-*		*Pestalotiopsis* sp. (16.7%)	*Wickerhamomyces anomalus* (31%)
	*Penicillium citreonigrum* (50%)			*Rhizopus stolonifer* (16.7%)	*Pichia exigua* (19%)
				*Pitomyces sacchari* (16.7%)	*Rhodotorula mucilaginosa* (19%)
				*Aspergillus fumigatus* (16.7%)	*Meyerozyma caribbica* (12.5%)
				*Penicillium griseofulvum* (16.7%)	*Torulaspora delbrueckii* (12.5%)
				*Didymella* sp. (16.5%)	*Candida orthopsilosis* (6%)
*Press water*	*Penicillium olsonii* (100%)			*Penicillium restrictum* (100%)	*Rhodotorula babjevae* (50%)
					*Meyerozyma caribbica* (50%)
*Sieving*	*-*		*-*	*-*	*Wickerhamomyces anomalus* (37.5%)
					*Rhodotorula mucilaginosa* (25%)
					*Naganishia diffluens* (12.5%)
					*Kwoniella heavenis* (6.3%)
					*Kazachstania unispora* (12.4%)
					*Candida orthopsilosis* (6.3%)
*Roasting*	*Alternaria infectoria* (33.3%)		*-*	*Stagonosporopsis* sp. (25%)	
	*Cladosporium sphaerospermum* (33.3%)			*Cladosporium sphaerospermum* (12.5%)	
	*Aspergillus flavus* (33.3%)			*Cladosporium cladosporioides* (12.5%)	
				*Cladosporium oxysporum* (12.5%)	
				*Rhizopus stolonifer* (12.5%)	
				*Arthinium* sp. (12.5%)	
				*Dothideales* sp. (12.5%)	

**Table 3:** Enumeration (log cfu/g) of different microbial groups isolated in “rale” processed in six cassava processing units during rainy, dry and both seasons. Values are presented as mean ± standard deviation (*n* = 3 for each season and *n* = 6 for both seasons). Different superscript letters represent significant differences (*p* < 0.05).

	Cassava Processing Units						
Microbes	*V ^1^*	*W ^1^*	*P ^1^*	*Z ^1^*	*C ^1^*	*J ^1^*	*p-Value ^2^*
**Rainy season (November 2020)**							
Yeast	*bdl*	*bdl*	*bdl*	*bdl*	*bdl*	*bdl*	*na*
Molds	0.36 ± 0.32	0.20 ± 0.35	1.33 ± 0.17	3.47 ± 2.29	1.53 ± 2.66	0.26 ± 0.24	0.1
*S. aureus*	*bdl*	2.33 ± 0.58	*bdl*	*bdl*	*bdl*	*bdl*	0.42
TBC	7.30 ± 4.59	*bdl*	*bdl*	*bdl*	3.33 ± 2.31	6.67 ± 3.21	0.054
*Enterobacteriaceae*	*bdl*	*bdl*	*bdl*	*bdl*	*bdl*	*bdl*	*na*
*Bacillus cereus*	*bdl*	*bdl*	*bdl*	*bdl*	*bdl*	*bdl*	*na*
**Dry season (August 2021)**							
Yeast	*bdl*	*bdl*	*bdl*	*bdl*	*bdl*	*bdl*	*na*
Molds	3.90 ± 0.53 ^a^	1.22 ± 1.10 ^a^	1.26 ± 0.12 ^a^	*bdl*	0.89 ± 0.25 ^a^	*bdl*	0.028 *
*S. aureus*	*bdl*	2.00 ± 0.01	*bdl*	*bdl*	*bdl*	*bdl*	0.42
TBC	1.83 ± 0.30	1.41 ± 0.17	1.75 ± 0.62	1.60 ± 0.11	1.67 ± 0.57	1.34 ± 0.57	0.66
*Enterobacteriaceae*	*bdl*	*bdl*	*bdl*	*bdl*	*bdl*	*bdl*	*na*
*Bacillus cereus*	*bdl*	*bdl*	*bdl*	*bdl*	2.33 ± 0.58	*bdl*	0.42
**All seasons (Rainy and Dry)**							
Yeast	*bdl*	*bdl*	*bdl*	*bdl*	*bdl*	*bdl*	*na*
Molds	2.13 ± 1.98	0.71 ± 0.92	1.29 ± 0.14	2.73 ± 1.66	1.21 ± 1.72	1.13 ± 0.97	0.15
*S. aureus*	*bdl*	2.17 ± 0.41	*bdl*	*bdl*	*bdl*	*bdl*	0.068
TBC	4.56 ± 4.18	1.70 ± 0.34	1.88 ± 0.41	1.80 ± 0.23	2.34 ± 1.86	4.17 ± 3.43	0.38
*Enterobacteriaceae*	*bdl*	*bdl*	*bdl*	*bdl*	*bdl*	*bdl*	*na*
*Bacillus cereus*	*bdl*	*bdl*	*bdl*	*bdl*	2.17 ± 0.41	*bdl*	0.42

Abbreviations: *bdl*—below the detection limit; *na*—not applicable; LAB—lactic acid bacteria; TBC—total bacterial count; cfu—colony forming units; ^1^ Mean (SD); ^2^ * *p* < 0.05.

**Table 4:** Molds (% of isolates) identified in “rale” processed in six cassava processing units during rainy and dry seasons.

		Rainy Season (November 2020)	Dry Season (August 2021)
		Mold isolates	
**Units**	*V*	*n.i.	*Aspergillus ruber* (29%)
			*Pithomyces sacchari* (43%)
			*Chaetomium globosum* (28%)
	*W*	*Penicillium purpureum* (17%)	*Pithomyces sacchari* (44%)
		*Alternaria* sp. (17%)	*Aspergillus penicillioides* (11%)
		*Aspergillus flavus* (17%)	*Aspergillus chevalieri* (22%)
		*Aspergillus niger* (17%)	*Fusarium solani* (11%)
		*Aureobasidium pullulans* (17%)	*Pleurotus ostreatus* (12%)
		*Cladosporium cladosporioides* (15%)	
	*P*	*Penicillium citreonigrum* (50%)	*Penicillium citrinum* (7%)
		*Fusarium oxysporum* (50%)	*Neopestalotiopsis egyptiaca* (7%)
			*Chaetomium globosum* (5%)
			*Pithomyces sacchari* (51%)
			*Alternaria alternata* (7%)
			*Pithomyces maydicus* (7%)
			*Paraphaeosphaeria michotii* (7%)
			*Aspergillus nidulans* (7%)
	*Z*	*Penicillium citreonigrum* (50%)	*-*
		*Penicillium ruber* (50%)	
	*C*	*Penicillium ruber* (67%)	*Phoma pereupyrena* (10%)
		*Trichoderma* sp. (33%)	*Pithomyces sacchari* (60%)
			*Aspergillus calidoustus* (10%)
			*Talaromyces* sp. (10%)
			*Aspergillus* sp. (10%)
	*J*	*Aspergillus niger* (14%)	*-*
		*Alternaria infectoria* (14%)	
		*Cladosporium sphaerospermum* (14%)	
		*Penicillium primulinum* (14%)	
		*Penicillium citreonigrum* (16%)	
		*Penicillium ochrochloron* (14%)	
		*Epicoccum* sp. (14%)	

Abbreviations: *n.i.—not identified.

**Table 5:** Enumeration (log cfu/g) of different microbial groups isolated in “rale” sold in five rural markets during rainy, dry and both seasons. Values are presented as mean ± standard deviation (*n* = 3 for each season and *n* = 6 for both seasons). Different superscript letters represent significant differences (*p* < 0.05).

Rural Markets						
Microbes	*AM ^1^*	*GB ^1^*	*MK ^1^*	*ES ^1^*	*MC ^1^*	*p-Value ^2^*
***Rainy season (November 2020)***						
Yeast	*bdl*	*bdl*	*bdl*	*bdl*	*bdl*	*na*
Molds	*bdl*	▪	*bdl*	▪	▪	*na*
*S. aureus*	*bdl*	*bdl*	2.01 ± 0.01	*bdl*	*bdl*	0.071
TBC	*bdl*	2.05 ± 0.09	*bdl*	*bdl*	*bdl*	0.41
*Enterobacteriaceae*	*bdl*	*bdl*	*bdl*	*bdl*	*bdl*	*na*
*Bacillus cereus*	*bdl*	*bdl*	*bdl*	*bdl*	*bdl*	*na*
**Dry season (August 2021)**						
Yeast	*bdl*	*bdl*	*bdl*	*bdl*	4.55 ± 0.55 ^a^	0.008 **
Molds	*bdl*	▪	0.26 ± 0.24 ^a^	▪	2.32 ± 0.36 ^a^	0.016 *
*S. aureus*	*bdl*	*bdl*	*bdl*	*bdl*	2.16 ± 0.28	0.41
TBC	2.46 ± 0.05 ^a^	2.54 ± 0.06 ^a^	1.71 ± 0.20 ^a^	2.06 ± 0.28 ^a^	1.86 ± 0.29 ^a^	0.023 *
*Enterobacteriaceae*	*bdl*	*bdl*	*bdl*	*bdl*	*bdl*	*na*
*Bacillus cereus*	2.01 ± 0.00 ^a^	*bdl*	*bdl*	*bdl*	2.33 ± 0.58 ^a^	0.050 *
**All seasons (rainy and dry)**						
Yeast	*bdl*	*bdl*	*bdl*	*bdl*	3.27 ± 1.44	0.012 *
Molds	*bdl*	▪	0.13 ± 0.21 ^a^	▪	1.16 ± 1.29 ^a^	0.045 *
*S. aureus*	*bdl*	*bdl*	2.00 ± 0.01	*bdl*	2.08 ± 0.19	0.24
TBC	2.23 ± 0.26 ^ab^	2.30 ± 0.28 ^a^	1.85 ± 0.20 ^b^	2.03 ± 0.18 ^ab^	1.93 ± 0.20 ^ab^	0.023 *
*Enterobacteriaceae*	*bdl*	*bdl*	*bdl*	*bdl*	*bdl*	*na*
*Bacillus cereus*	*bdl*	*bdl*	*bdl*	*bdl*	2.17 ± 0.41	0.063

Abbreviations: *bdl*—below the detection limit; *na*—not applicable; LAB—lactic acid bacteria; TBC—total bacterial count; **▪**—less than two colonies/plate (10^−1^); cfu— colony forming units; ^1^ Mean (SD); ^2^ * *p* < 0.05; ** *p* < 0.01.

**Table 6:** Molds (% of isolates) identified in “rale” samples collected in five rural markets during rainy and dry seasons.

		Rainy Season (November 2020)	Dry Season (August 2021)
		Mold Isolates	
**Markets**	*AM*	*Talaromyces amestolkiae* (100%)	*bdl*
	*GB*	*Fusarium petroliphilum* (100%)	*Talaromyces* sp. (100%)
	*MK*	*bdl*	*Pithomyces sacchari* (80%)
			*Aspergillus shendaweii* (20%)
	*ES*	*bdl*	*Pithomyces sacchari* (100%)
	*MC*	*Fusarium solani* (50%)	*Pithomyces sacchari* (25%)
		*Rhizopus oryzae* (50%)	*Aspergillus calidoustus* (25%)
			*Trematosphaeria grisea* (25%)
			*Pithomyces chartarum* (25%)

Abbreviations: *bdl*—below the detection limit.

## Data Availability

The raw data supporting the conclusions of this article will be made available by the authors on request.
